# Evaluation of microbial diversity in the formation water of the producer and marginal wells in bokaro coal field

**DOI:** 10.1038/s41598-024-61996-2

**Published:** 2024-11-28

**Authors:** Nishi Sahu, Meeta Lavania, Dipanjana Banerjee, Mansi Chawla, Banwari Lal

**Affiliations:** Microbial Biotechnology, Environmental and Industrial Biotechnology Division, The Energy and Resources Institute (TERI), New Delhi, India

**Keywords:** Biogenic Coalbed Methane, Microbial community, Producing wells, Marginal wells, Environmental parameters, Microbiology, Environmental sciences

## Abstract

The rise in global energy demand has prompted research on developing strategies for transforming conventional nonrenewable sources to cleaner fuels. Biogenic methane production is a promising source that caters to increasing energy demands. Therefore, research to enhance their production is of great importance. Implementation of successful enhancement strategies requires knowledge of the factors impacting coalbed methane production. The microbial diversity of the formation water in coal seams is the crucial parameter influencing biomethane production. This study explores microbial diversity in the Producing and Marginal wells of Bokaro, India, intending to understand the potential application of microbial-enhanced coalbed methane technology in the marginal wells of this reservoir. The high throughput sequencing analysis revealed the presence of both archaeal and bacterial groups in both well types. The result showed significant differences in the diversity of the samples from the two well groups, suggesting the immense role played by the microbes in producing methane gas. Random forest analysis shows genera *Gelria, Methanothermobacter, Thaurea, Youngiibacter,* and *Proteiniclasticum* in the Producing wells while *Roseomonas, Rhodobacter, Mycobacterium, Methylobacter,* and *Bosea* in the Marginal wells as the significant contributor in differentiating the overall diversity between the wells of Bokaro. The current study is the first to show microbial uniqueness in coalbed methane wells based on gas production efficiency. It also explores the role of physicochemical factors in framing microbial community structure in the wells. The results provide salient information that will help better understand the impact of microbial diversity on the production of coalbed methane wells of studied coal seams. This knowledge will further aid in exploring the prospects of microbial-enhanced methane in the Marginal wells.

## Introduction

Regardless of efforts made globally to lessen reliance on fossil fuels, coal remains a major fuel for energy production. Since coal is the primary source of CO_2_ emissions and electricity generation, switching to low-carbon energy systems presents a unique challenge. Coal Bed Methane (CBM) is an unconventional form of natural gas formed inside coal seams^1^. CBM is a clean form of energy; therefore, its development and utilization carry great social and economic benefits and provide a way forward for the global energy transition^1^. Considering the surge in energy demand and environmental perspective, CBM is a better alternative to fulfill domestic and industrial energy demands^2^.

Production of methane from coal beds is biogenic and thermogenic^3^. Biogenic methane production involves complex metabolism performed by a consortium of indigenous microbes^4^. The coal seam ecosystem is an example of synchronizing hydrolyzing and fermenting microbes with methanogens. The methane generation process involves the degeneration of complex hydrocarbons to lower molecular weight organic compounds, which act as a substrate for the methanogens and are finally converted into methane gas^5^. Methanogens can be divided into three categories according to the metabolic pathway followed in methane production. Hydrogenotrophic methanogens utilize hydrogen as an electron donor to reduce carbon dioxide to methane, e.g., *Methanobacterium.* The acetotrophic methanogens convert acetate to methane and CO_2,_ e.g., *Methanosarcina* and *Methanosaeta.* Lastly, the methylotrophic methanogens convert methanol and methylamine into methane^3^.

Recently, the development of biogenic CBM has picked up and is gathering more attention. Researchers are focusing on microbial diversity to understand the microbial ecosystem and their functioning in the production of CBM^6^. A recent study explored seasonal variation in coal bed water in China and reported the prevalence of phyla *Proteobacteria, Bacteroidetes,* and *Firmicutes*^3^. The comparative analysis of coal bed diversity of Gunnedah, Sydney, and Surat coals shows the abundance of *Firmicutes, Proteobacteria, Euryarchaeota, Bacteroidetes,* and *Actinobacteria*^7^. Another study exploring the Jharia coal mine's formation water diversity revealed *Proteobacteria, Bacteroidetes, Actinobacteria, Verrucomicrobia,* and *Firmicutes* as the dominant phyla in the formation water^8^. These studies established the significant role of these members in the production of biogenic methane by degradation of complex polymers and providing substrate for methane production.

Worldwide efforts are being made to enhance the production of biogenic CBM. Coal provides over 58% of India's energy demands, followed by hydrocarbons at 38% and nuclear and hydroelectric power at 4% each. The high reliance creates immense pressure to limit the coal dependency^9^. A recent report suggested that harnessing 10% of coal bed methane reserves can cut India's energy import bill by two billion US dollars (https://energy.economictimes.indiatimes.com/news/coal/harnesing-10-of-coal-bed-methane-reserves-can-cut-indias-energy-import-bill-by-2-billion-experts/100237602). Therefore, a deeper understanding of the microbial diversity and environmental factors that drives the CBM production process is essential. Despite the various research in this area, there is a void in understanding variation in microbial diversity of CBM wells differing in their gas production^10^. The present study utilizes metagenomics to decipher microbial communities in the Producing and the Marginal groups of CBM well in Bokaro, India. The present investigation is the first to demonstrate microbial distinctiveness in the CBM wells according to the gas production performance. The study will facilitate a better understanding of the roles of the microbes in the production of biogenic methane, which may further help in implementing field jobs for its augmentation in the wells.

## Materials and methods

### Sample area description and collection

**Figure 1:**
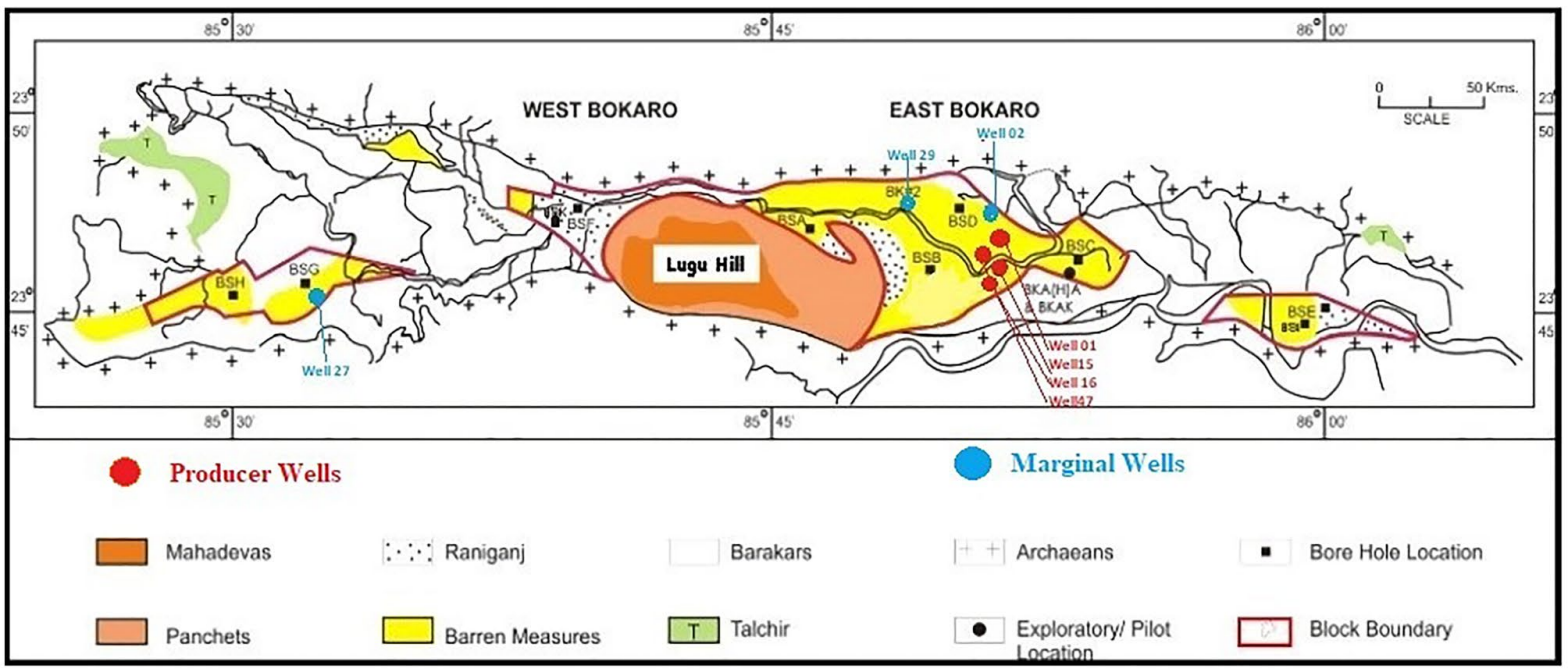
Geographical location of the study area, sampling points, and distribution of samples. The red dots represent the Producer wells, and the blue dots represent the Marginal wells

The formation water and coal samples were collected from CBM wells, namely Well01, Well02, Well15, Well16, Well27, Well29, and Well47. The well belongs to the Bokaro coal bed methane block of Oil and Natural Gas Corp. in eastern India. The location and grouping details of the samples are shown in Table 1 and Fig. 1. The Bokaro Block, extending over 95 km^2^, consists of three disconnected sectors. Central Patch (Patch-A covering 66 km^2^ area), Western Patch (Patch B covering 18 km^2^ area), and Eastern Patch (Patch-C covering 11 km^2^ area). The Central Patch comprises three distinct geographic areas viz. (i) Saram-Gumia in the adjoining eastern part of the Lugu hill, (ii) Jarangdih Deep beyond the Saram-Gumia area in the further east separated by the Bokaro River, and (iii) Daniya to the west of Lugu hill. The western patch lies in the Hesagara-Kasikhap area, whereas the Eastern Patch lies in the Chalkari-Phusro area.

**Table 1:** Location of Bokaro samples and grouping information.

Well number	Location	Group
WELL#01	23°46′19.53″N 85°50′49.30″ E	Producer
WELL#15	23°45′33.82″N 85° 50′ 06.83″ E	Producer
WELL#16	23°45′53.52″N 85° 50′35.43″E	Producer
WELL#47	23°45′53.55″N 85° 50′35.42″E	Producer
WELL#02	23°46′22.5″ N 85° 52′44.6" E	Marginal
WELL#27	23°45′30.52″N 85° 31′13.47″E	Marginal
WELL#29	23°46′04.71″N 85° 50′58.39″E	Marginal

The Bokaro Coalfield forms a part of the Damodar Valley coalfields and is situated in the Bokaro and Hazaribagh districts of Jharkhand. This coalfield is one of the country's major producers of medium-cooking blendable coals. Lugu Hill, a major topographic high of the area, divides the coalfield into two parts. East Bokaro Coalfield and West Bokaro Coalfield. The Damodar River flows along the southern margin of East Bokaro Coalfield, and the Bokaro River forms the main drainage of West Bokaro Coalfield.

The samples are grouped according to the wells' annual gas production. Wells with gas production > 500 m^3^/d belong to the Producer groups, and the wells with production < 500 m^3^/d belong to the Marginal group.

The sampling of all the wells was done in April 2022. The sample collection device consisted of a 10 cm diameter, 1 m long polyvinyl chloride pipe sealed at the bottom and capped with an open steel mesh at the top. The pipe and mesh enclosure were sterilized with a bleach solution before being lowered to the well's bottom on a wireline^11^. Samples were collected in triplicate in 1000 ml of anaerobic sterilized serum bottles and analyzed for pH and conductivity on site. All the samples were transported to the laboratory within 48 h at 4 °C and processed immediately for activity measurement and microbial analysis^12^.

The indigenous coal samples of a CBM well were obtained from the core house and transferred in sterile, airtight containers.

### Physio-chemical analysis of formation water and coal

Physio-chemical analysis of the formation water samples from the Bokaro CBM well was performed for pH, Total dissolved solids (TDS), Total suspended solids (TSS), and Electrical conductivity using the pH conductivity meter instrument (Seven Compact pH meter S220, Metler Toledo, USA). Heavy metals such as arsenic, cadmium, chromium, copper, zinc, nickel, silver, and total iron were estimated per the standard methods mentioned in Table 2. The analysis of the water samples was done using atomic absorption spectroscopy (Thermo Scientific Model-AA301, USA). The presence of cation and anions calcium, sodium, flouride, and sulphate was examined following the standard method mentioned in Table 2. The detailed evaluation of coal in terms of proximate and ultimate analysis, such as ash, moisture, volatile matter, and fixed carbon, along with the specific carbon, hydrogen, nitrogen, sulphur, and oxygen content, was estimated as per the standard guidelines Table 3. The calorific value of the coal sample was analyzed using a bomb calorimeter (Ferrotek equipment FE-255A, Ghaziabad, India).

**Table 2:** Physico-chemical parameters of formation water collected from Bokaro wells.

Parameters	Test Method	WELL#02	WELL#29	WELL#27	WELL#01	WELL#15	WELL#16	WELL#47
pH	Metler Toledo instrument	7.82	7.87	7.56	7.31	7.30	7.14	7.33
Conductivity (μS/cm)	Metler Toledo instrument	1632	640.3	2598	8925	7507	7610	6590
TDS (mg/L)	Metler Toledo instrument	865	320.3	1286	4438	3731	3786	3238
TSS (ppm)	Metler Toledo instrument	75	50	120	50	70	310	140
		Results (mg/L)						
Sodium	IS 3025 (Pt-45):1993	2.8	11.8	59.2	2472.25	2428.75	2469.5	2027.25
Calcium	IS 3025 (Pt-40):1991	7.21	51.30	35.4	27.7	15.39	29.24	29.24
Flouride	IS 3025 (Pt-60):2008	0.23	0.43	3.07	5.82	1.38	1.2	3.56
Sulphate (as SO_4_)	IS 3025 (Pt-24):1986	1.87	3.12	4.7	3.5	10.6	13.1	5.7
Silver	IS 3025 (Pt-65):2014	ND < 0.05	ND < 0.05	BLQ	ND (< 0.002)	ND (< 0.002)	ND (< 0.002)	ND (< 0.002)
Zinc	IS 3025 (Pt-65):2014	ND < 0.05	ND < 0.05	0.06	ND (< 0.002)	0.028	0.015	ND (< 0.002)
Copper	IS 3025 (Pt-65):2014	ND < 0.02	0.13	BLQ	ND (< 0.002)	ND (< 0.002)	ND (< 0.002)	ND (< 0.002)
Iron	IS 3025 (Pt-65):2014	1.24	30.8	13.39	1.86	3.4	6.26	0.237
Manganese	IS 3025 (Pt-65):2014	ND < 0.02	ND < 0.02	0.263	ND (< 0.002)	0.047	0.066	0.011
Magnesium	APHA-3500-Mg (B)	5.71	103.03	20.52	4.66	6.53	5.59	5.59
Nickel	IS 3025 (Pt-65):2014	ND < 0.03	ND < 0.03	0.006	ND (< 0.002)	ND (< 0.002)	ND (< 0.002)	ND (< 0.002)
Cadmium	IS 3025 (Pt-65):2014	ND < 0.04	0.04	BLQ	ND (< 0.0001)	ND (< 0.0001)	ND (< 0.0001)	ND (< 0.0001)
Lead	IS 3025 (Pt-65):2014	ND < 0.01	ND < 0.01	ND (< 0.002)	ND (< 0.002)	0.006	ND (< 0.002)	ND (< 0.002)
Arsenic	IS 3025 (Pt-65):2014	ND < 0.01	ND < 0.01	ND (< 0.002)	ND (< 0.002)	ND (< 0.002)	ND (< 0.002)	ND (< 0.002)
Chromium	IS 3025 (Pt-65):2014	ND < 0.01	ND < 0.01	ND < 0.01	0.023	0.02	0.015	ND (< 0.002)

**Table 3:** Physicochemical parameters of representative coal samples.

Test parameters	Method of measurement	WELL#02	WELL#29	WELL#27	WELL#01	WELL#15	WELL#16	WELL#47
Ultimate analysis								
Carbon (% by mass)	IS:1350 (P-4)Sec-1:1974	53.19	57.15	61.78	67.12	62.48	54.14	60.47
Hydrogen (% by mass)	IS:1350 (P-4)Sec-2:1975	3.96	3.08	4.09	3.96	4.06	3.24	3.45
Nitrogen (% by mass)	IS:1350 (P-3) :1969	1.18	1.05	1.21	1.21	1.5	1.73	1.32
Sulphur (% by mass)	IS:1350 (P-4)Sec-1:1974	0.43	0.34	0.41	0.36	0.31	0.67	0.49
Mineral Matter (% by mass)	IS:1350 (Pt-1)1984,Reff-2007 guidelines	38.81	36.04	29.64	21.45	27.31	35.47	27.21
Moisture (% by mass)	IS:1350 (Pt-1)1984,Reff-2007 guidelines	0.18	0.24	0.17	0.13	0.22	0.18	0.16
Oxygen Content (% by mass)	By Difference	2.25	2.1	2.7	5.77	4.12	4.57	6.7
Proximate analysis								
Ash (% by mass)	IS:1350 (P-2) : 1970	35.28	22.35	24.35	23.85	36.3	32.65	30.7
Inherent Moisture (% by mass)	IS:1350 (P-1) : 1984	0.06	0.06	0.09	0.008	0.05	0.17	0.05
Moisture content (% by mass)	IS:1350 (P-1) : 1984	0.24	0.24	0.17	0.13	0.12	0.07	0.07
Volatile matter (% by mass)	IS:1350 (P-1) : 1954	22.18	21.08	22.92	21.45	22.94	22.78	23.04
Fixed Carbon by Difference (% by mass)	IS:1350 (P-1) : 1984	42.36	56.27	52.47	54.57	40.59	44.33	46.14
Gross Calorific Value (Kcal/kg)	IS:1350 (P-2) : 1970	5006	5347	6147	6387	6259	5124	6098

### DNA extraction from the formation water and amplicon sequencing

In order to assess the native microbial diversity of the formation water of CBM wells, genomic DNA extraction was performed. The formation water from each well was collected in triplicate for the extraction of microbial DNA. For this, the triplicate water samples from each well were pooled together, and then 500 ml of pooled water sample was passed through a membrane filter (90 mm, 0.22 μm). The formation water contained suspended coal particles. To avoid losing those microorganisms attached to coal particles, all that was retained on the filter was then processed for DNA extraction using DNeasy PowerWater Kit (Qiagen, Germany). DNA was extracted as per the manufacturer's protocol. Following extraction, DNA samples were quantified and evaluated. The extracted DNA sample with good quality (A260/A280: 1.8–2.0) and concentrations (more than 50 ng/μl) was taken up further for sequencing^11^.

Polymerase chain reaction (PCR) amplification was done using primers for 16S rRNA (338F: ACTCCTACGGGAGGCAGCA, 806R: GGACTACHVGGGTWTCTAAT, targeting the V3-V4 region)^13^. Amplicon libraries were prepared with high-quality DNA and Nextera Index Kit 16S metagenomic sequencing library preparation protocol. Libraries were sequenced using the Illumina MiSeq platform with 2*300 base-pair chemistry at Medgenome Pvt. Ltd., Bangalore, India.

### Sequence pre-processing, OTU picking, and downstream processing

FASTQC tool v 0.11.8 (Babraham bioinformatics) verified the quality of sequences after demultiplexing and adaptor/primer/ barcodes sequence removal from raw reads. Merging of paired-end reads of each sample was carried out using FLASH v 1.2.11 software^14^. Quantitative Insights Into Microbial Ecology, QIIME 2 standard protocol was followed^15^. For sequence quality control, the denoise_paired action in the dada2 plugin was performed. This did quality filtering, chimera checking, and paired-end read joining. Operational Taxonomic Units (OTU) picking was done against SILVA db version 138 database^14,15^. Cumulative sum scaling^17^, low variance, and low count filtering^18^ were carried out before downstream analysis. These pre-processing steps enabled the avoidance of sequencing depth bias for better comparative analysis.

### Metagenomic analysis-culture-independent approach

Comparative analysis of microbiomes was done at alpha and beta diversity levels. At the alpha level, the analysis was done using the Chao diversity index. The pie charts were generated using Microbiome Analyst software^19^. Core Microbiome and Random Forest analysis in the Microbiome Analysts platform were used to identify signature microbes in the river systems^19^. Canonical Correspondence analysis (CCA) was conducted to determine the relationship between physicochemical factors and normalized abundances of major taxonomic groups (genus) using PAST v4.03 software^20^.

## Results and discussion

### Physico-chemical analysis of formation water and coal

A physio-chemical analysis of the formation water collected for this study is shown in Table 2. The pH ranges from 7.14 to 7.87. The TDS of the formation water ranges from 320 to 4438 mg/l. Also, sulphate, chloride, fluoride, and iron were found (Table 2). The physicochemical analysis shows differences in various parameters in the formation water of two groups. The analysis shows high sodium, TDS, and conductivity in the Producer wells. Previous studies have shown that high TDS, salinity, and conductivity are indicative of high coal bed methane in the wells^21–23^. The gas phase desorption of methane can be accelerated by salinity, or the TDS in the formation water, as it can compete with methane for coal surface adsorption sites^24^. The concentration of sodium was found to be significantly higher in the Producer wells. This finding relates to another study that reported high sodium concentrations in the formation water of CBM wells^25^. Research reveals that high sodium and low calcium, magnesium, and sulfate concentrations in groundwater typically point to a high CBM enrichment potential, which is favorable for high production outcomes^26^. High sodium and TDS in the Producing wells may contribute to high CBM production in the wells. Further, most of the heavy metals in the samples were found below the detection limit or in very low concentrations, suggesting ambient conditions for the growth of the microbes.

The proximate and ultimate analysis of coal samples of the seams from the Bokaro CBM wells was carried out (Table 3). The coal contains low moisture (0.07–0.24%), medium ash (22.35–36.3%) with 21.08–23.04% volatile matter, and 40.59–56.27% fixed carbon. Ultimate analysis results indicated low sulfur content, carbon ranging from 53.19 to 67.12%, and hydrogen at 3.08–4.09% (Table 3). The calorific value of the coal sample was found to be 5006–6387 kcal/kg. The analysis of all the coal samples indicates that the ASTM rank of the coal is high volatile 'A' bituminous (HVAB). The analysis showed the same category of coal in all the selected wells.

### General statistics for 16S rRNA sequencing and microbial diversity analysis in the producer wells and marginal wells

After pre-processing and quality filtering, the resulting library size included a total of 2,435,998 sequences. A total library size of 274,498 sequences was further utilized for analysis after OTU picking. The average library size of samples was 39,214, which was further rarefied to the minimum library size.

The rarefaction curve based on the observed OTU shows that the produced sequence adequately represents the present microbial communities in the wells. The rarefaction curve also depicts higher species richness in Well 47 in Producing wells, while in the Marginal wells, the highest species richness was found in Well 27 (Figure S1).

**Figure 2:**
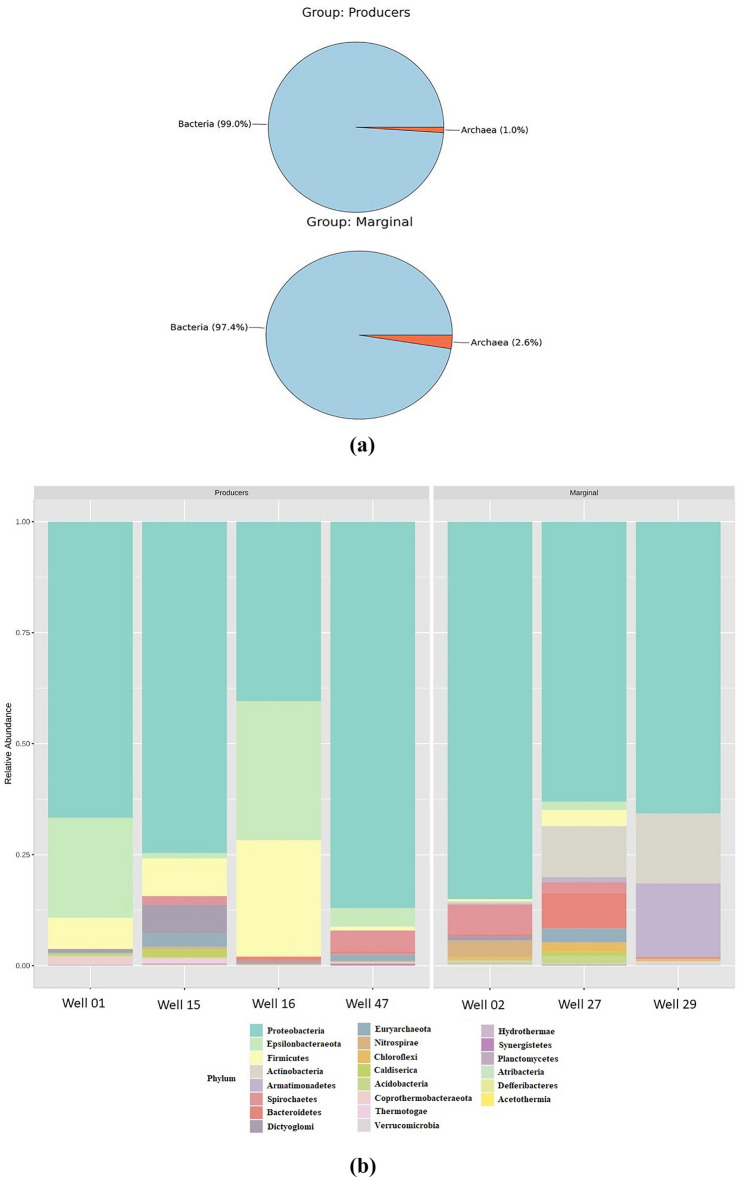
Microbial abundance of the taxonomic groups in the Producer and Marginal wells (**a**) Pie charts represent abundance at the domain level, (**b**) Stacked bar plot showing phylum level abundance.

**Figure 3:**
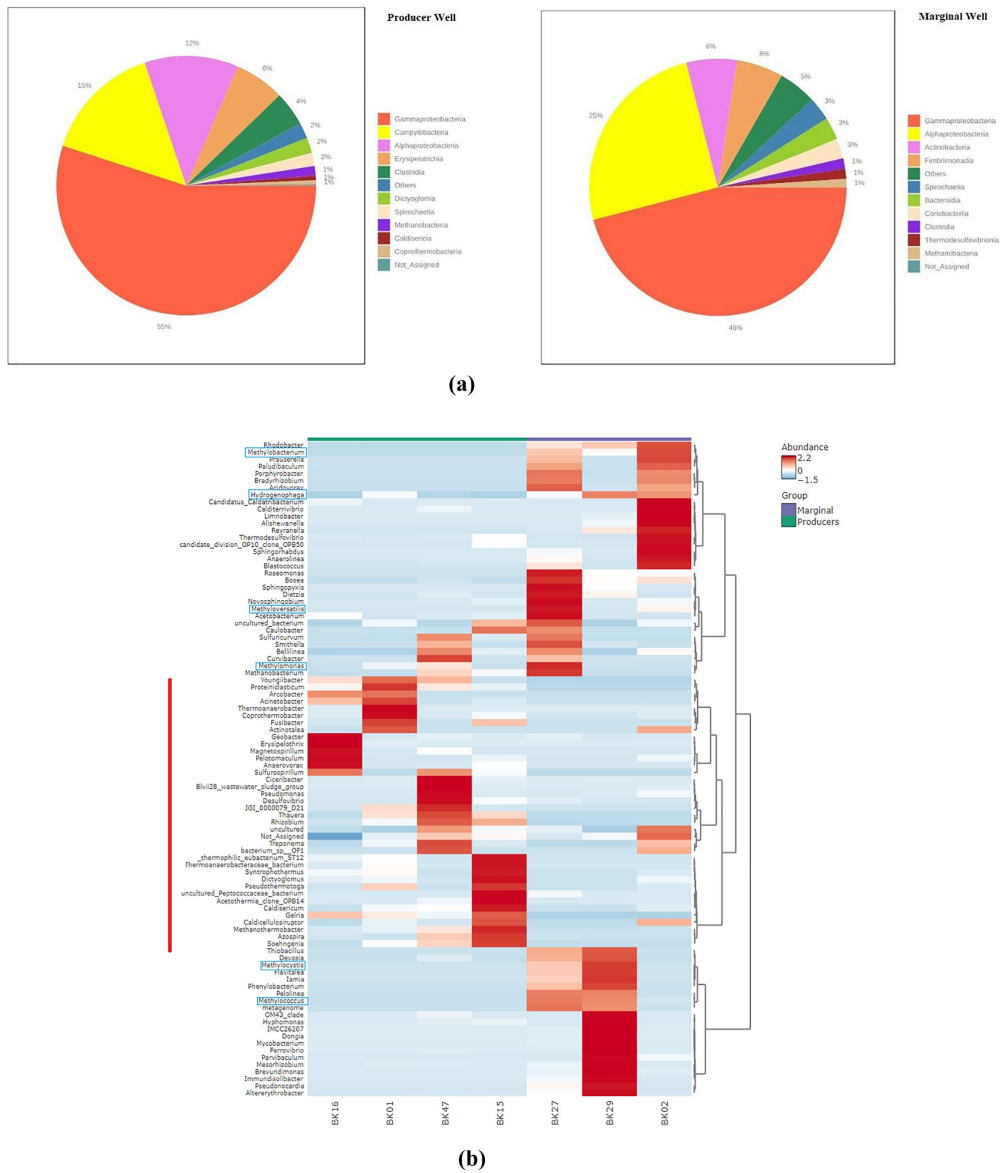
(**a**) Pie charts depicting microbial abundance at the class level in Producer and Marginal well, (**b**) Heatmap showing abundance of microbes in Producer and Marginal wells at the genus level. The red line indicates abundant genera in Producer wells. The blue box indicates microbes that consume methane and hydrogen.

The amplicon sequencing reveals the abundance of the diverse microbiome in the formation water of Producers and Marginal wells. At the domain level, both bacterial and archaeal groups were present (Fig. 2a). The Producer wells show an abundance of *Bacteria*—at 99% and *Archaea* at 1%, whereas the Marginal wells show an abundance of *Bacteria*—at 97.4% and *Archaea* at 2.6%. The archaeal groups comprise members of the class *Methanobacteria* belonging to the *Euryarchaeota* phylum, suggesting hydrogenotrophic as the primary pathway for generating biogenic methane in the wells^27^. According to the sequence read classification, the relative abundance analysis shows the variance in the abundance of dominant phyla in the two groups (Fig. 2b). The dominant phyla in the Producer group were *Proteobacteria (*recently named *Pseudomonadota), Epsilonbacteraeota, Firmicutes (*recently named *Bacillota), Dictyoglomi (*recently named *Dictyoglomerota),* and *Spirochaetes (*recently named *Spirochaetota)*. In contrast, the dominant members in the Marginal groups were *Proteobacteria, Actinobacteria (* recently named *Actinomycetota), Armanimondetes, Bacteroidetes (*recently named *Bacteroidota ),* and *Spirochaetes*. Phyla *Proteobacteria, Firmicutes*, and *Bacteroidetes* are the commonly identified bacterial phyla in the anaerobic digestive systems^28^. At the class level, *Proteobacteria* consists of *Gammaproteobacteria, Alphaproteobacteria*, and *Deltaproteobacteria* (Fig. 3a). Studies have shown *Gammaproteobacteria's* important role in mediating the utilization of methyl-, sulfur- and petroleum organic compounds in deep ocean hydrothermal plumes^29^. Members of *Deltaproteobacteria* have been studied for their hydrocarbon-degrading capabilities in anaerobic conditions^30^. Figure 2b shows the abundance of phylum *Firmicutes* in Producer wells. This phylum comprises members of the class *Clostridia, Erysipelotrichia,* and *BRH_c20a*. Members of the class *Clostridia* are known to be involved in hydrogen-producing mechanisms^31,32^. Hydrogen acts as the limiting factor in the hydrogenotrophic methanogenesis pathway; therefore, the presence of these members may immensely impact the gas production in the wells^33^. Members of class *Dictyoglomus* are abundantly present in producing wells. *Dictyoglomus* are extremely thermophilic, chemoorganotrophic, and obligate anaerobes^34^. Members of class *Spirochaetia* are involved in syntrophic acetate oxidation in anaerobic methanogenesis^35^. These variations in microbial diversity and other parameters may contribute to the difference in biogenic methane production. Previous studies have shown higher coal methanogenesis in samples with a high abundance of *Firmicutes.* Thus suggesting that coal methanogenesis is unlikely to be limited by methanogen biomass but rather by the activation and degradation of coal constituents^36^. The phylum *Actinobacteria* has been recently reported for their significance as purportedly resistant organic matter decomposers, particularly lignocellulose, and consequently, their capacity to aid in the creation of bio-based goods (energy and materials) while lowering carbon emissions has been taken into account^37^. The archaeal phyla *Euryarchaeota* mainly consists of class *Methanobacteria*, thus showing hydrogenotrophic production of methane as the prominent way of methanogenesis. This group of methanogens utilizes hydrogen as an electron donor to reduce carbon dioxide to methane^38^.

The differential abundance of microbes is further supported by the heatmap analysis at the genus level (Fig. 3b). The red line marks the higher abundance of genus members in the Producing wells. Genus *Youngiibacter* belongs to *Firmicutes* phylum, abundant in Producer wells, and is a newly described genus of the family *Clostridiaceae*. It's a strictly anaerobic bacteria that ferments a range of carbohydrates to ethanol, formate, acetate, and CO_2_^39^. Previous studies have reported *Rhizobium* in coal and formation water samples^40,41^. Studies have also shown that *Rhizobium* members have phenol and trichloroethene degrading capabilities^42^. The members of the genus *Sulfurospirillum, Proteiniclasticum*, produce hydrogen and acetate as bi-products of their metabolism^43,44^, thus responsible for methane production enhancement. Genus *Soehngenia, Proteiniclasticum*, and *Gelria* belong to the class *Clostridia*. *Soenhgenia* is a fermenting bacterium often isolated from petroleum reservoirs^45^. *Gelria* is an anaerobic thermophilic bacteria obligatory syntrophic bacteria; few members are often isolated from was isolated from a propionate-oxidizing methanogenic enrichment culture^46^. Genus *JGI_0000079_D21, abundant* in Producer wells, is associated with the degradation of phenols and N-heterocyclic compounds in anaerobic digestion^47^. Members of *Coprothermobacter* grow in a protein-rich environment and are associated with hydrogen and methane production. They also have syntrophic relationships with hydrogenotrophic methanogenic archaea^48^. *Methanothermobacter* in the Producing wells shows the presence of thermophilic methanogens, thus carrying methanogenesis at higher temperatures^49^. The analysis also reveals the abundance of *Methyloversatilis, Methylococcus, Methylocystis, and Methylobacterium* in the Marginal samples. These members use methane as a substrate for their metabolism^50^. Their abundance in the Marginal group might significantly contribute to the lesser methane production in the wells. Genus *Hydrogenphaga* are abundant in Marginal wells and are hydrogen-oxidizing bacteria^51^. Therefore, their abundance may limit the availability of hydrogen to methanogens. A previous study has also reported their presence in CBM wells. However, their function in the wells needs more exploration^52^.

The heatmap analysis also reveals variation in the relative abundance of microbial groups among the wells of the Producer group. Geographical variations, location, nutrient availability, and other factors impact the relative abundance of microbes in the formation water of coal beds^53,54^. The analysis also revealed variations in the abundance of methanogens (*Methanobacterium* and *Methanothermobacter*) among the wells of the Producer group. The variation in methanogen abundance demonstrates how, in addition to methanogen abundance, various other factors also control CBM production. Therefore, CBM production in the wells is unlikely to be limited by methanogenic mass^55^.

### Alpha and Beta Diversity Analysis

**Figure 4:**
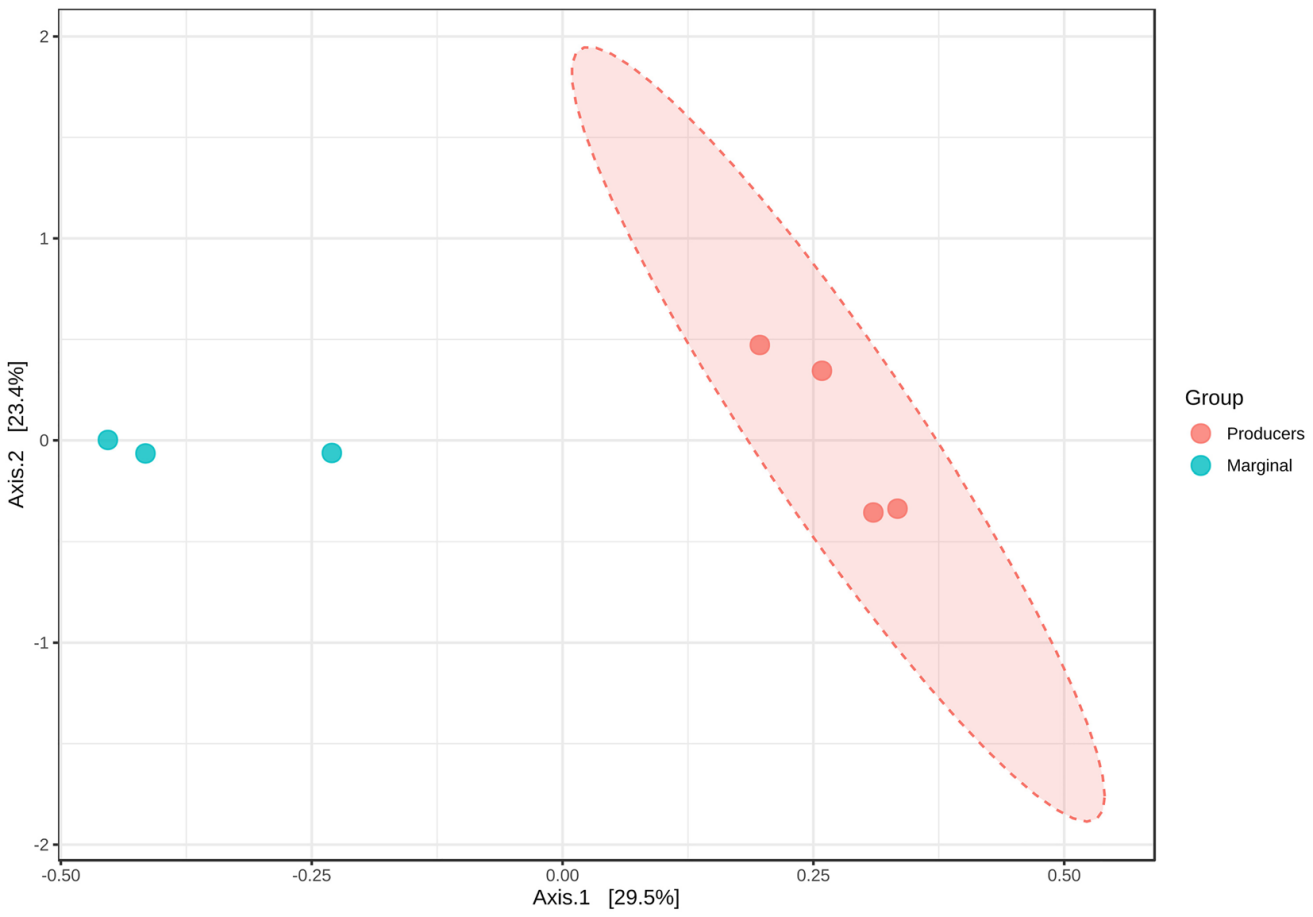
Beta diversity evaluation at the genus level: Principal Coordinate Analysis (PCoA) based on BrayCurtis metrics shows the dissimilarity of microbial communities in Producer and Marginal wells. [ANOSIM] R: 0.318; *p* value < 0.05.

The alpha level diversity shows the highest abundance and richness in Well#47 in the Producer wells, and in the case of Marginal wells, the highest abundance and richness was found in Well#27 (Figure S2). Beta diversity projection on the PCoA plot revealed significantly different communities between the Producers and Marginal wells at the genus level (Fig. 4, ANOSIM, p-value < 0.05). The significant variance supports the existence of diverse microbes in both wells. This signifies microbial communities' role in producing methane from the well. The distinctiveness in the microbial diversity in the two groups is also supported by the dendrogram analysis, which shows the samples of the two groups belong to two different clusters (Figure S3). Previous studies have demonstrated distinct microbial communities residing in dissimilar hydrological areas of southern Qinshui Basin coal reservoirs^56^. Another study revealed seasonal variation in the microbial communities in the Erlian basin, China^3^.

### Differentially abundant taxa in the producer and the marginal wells

**Figure 5:**
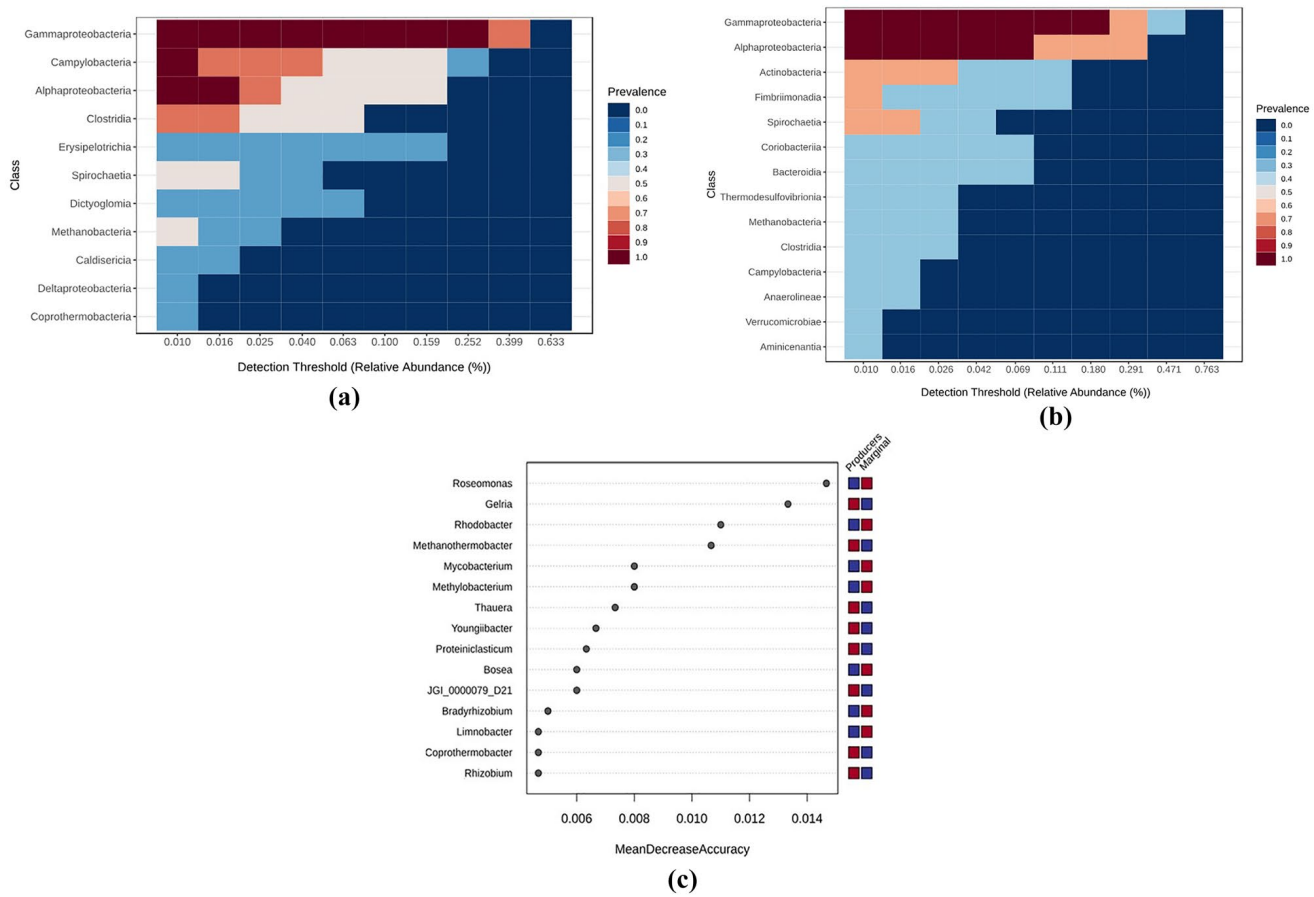
Core Microbiome Analysis at the class level in (**a**) Producer well and (**b**) Marginal wells. (**c**) Random Forest Analysis. The top 10 genera with the highest distinct abundance between both well groups are listed. Red fields show a high abundance, and blue shows a low abundance of the particular genus in the well group.

The differences between the microbial communities can be understood by analysis of the differentiating members of the core microbiome. A core microbiome represents those genomes or genetic markers common to all the samples studied and is critical to the genetic functions and composition of the microbial communities^57^. Therefore, it is crucial to identify the community structures and ecological processes of the core microbiome in the CBM wells (Fig. 5a, b). At the class level, *Gammaproteobacteria, Campylobacteria, Alphaproteobacteria, Clostridia, Spirochaetia,* and *Methanobacteria* were present in the Producer well groups. In contrast, the Marginal group shows class *Gammaproteobacteria, Alphaproteobacteria, Actinobacteria, Fimbriimonadia,* and *Spirochaetia*. Microbes belonging to these classes are widely reported in Indian coal bed wells^8,11^. The analysis of the core microbiome suggests the presence of hydrogen-producing (*Clostridia*), syntrophic acetate oxidative microbes (*Spirochaetia*)^58^, and hydrogenotrophic methanogens in all the samples of the Producer groups. Research has shown that syntrophic acetate oxidative microbes play a key role in hydrogenotrophic methanogenesis^59^. The analysis shows the importance of synergy among these microbes and their critical role in the process of methanogenesis in the CBM wells.

Random forest analysis shows a significant association of microbes with the Producing and the Marginal wells (Fig. 5c)*. Gelria, Methanothermobacter, Thaurea, Proteiniclasticum, Youngiibacter, JGI_0000079_D21, Coprothermobacter,* and *Rhizobium* were significantly associated with Producing wells. The Marginal wells were found to be significantly associated with the genera *Roseomonas, Rhodobacter, Mycobacterium, Methylobacterium, Bosea, Bradyrhizobium,* and *Limnobacter.* The role of Rhodobacter and Roseomonas member*s* is studied in modulating the anaerobic digestion of different substrates^60,61^. Members of *Methylobacterium* use methane as a substrate for their metabolism^50^. Random forest analysis further strengthens the relationship between microbial members belonging to *Clostridia* and *Methanothermobacter* (methanogens) members with the Producer group. These findings corroborate the previous studies, which show an abundance of these microbial groups in methane-producing systems^62^^63^.

### Relationship between microbial community and physicochemical parameters of wells

**Figure 6:**
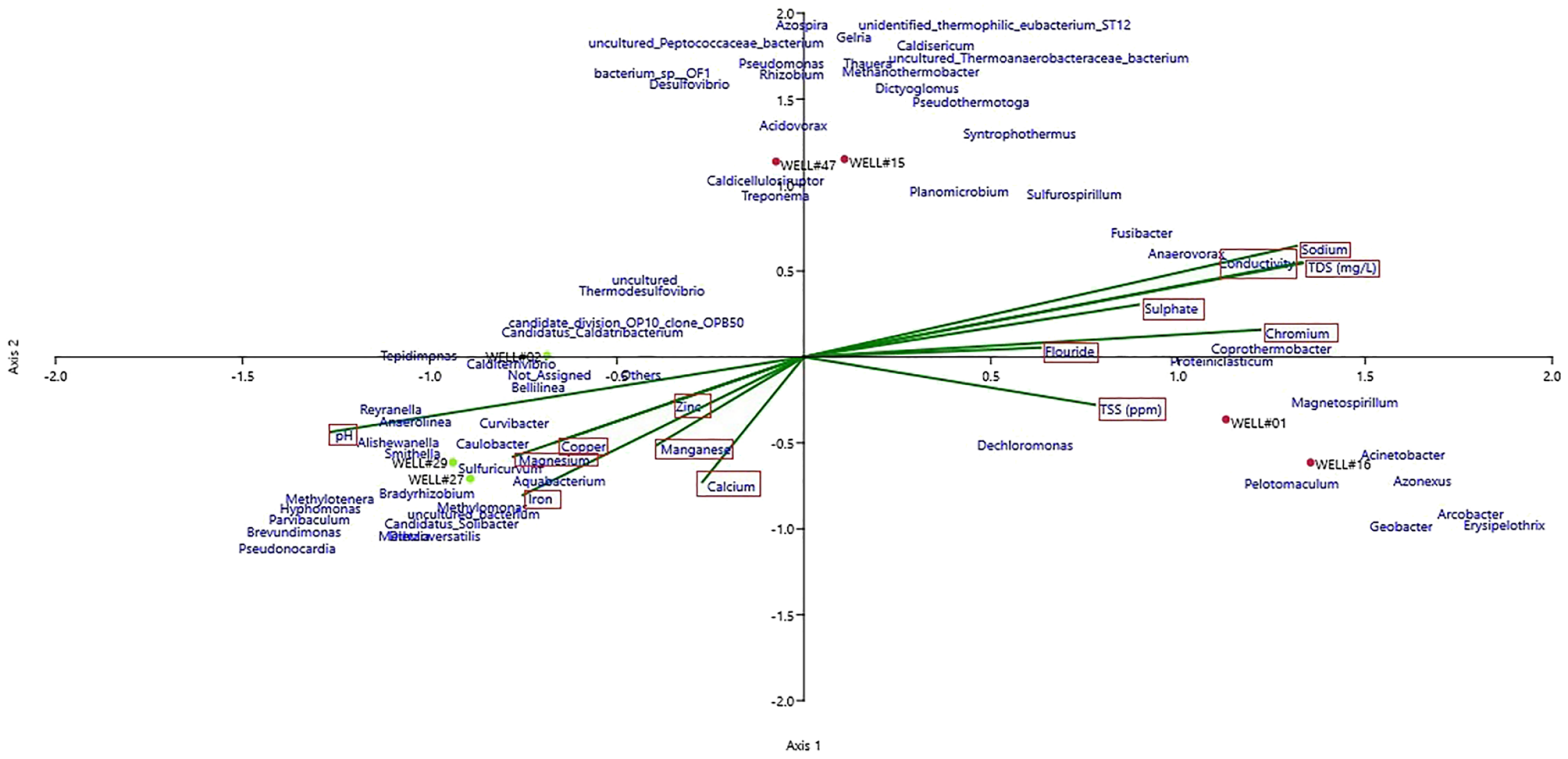
Canonical correspondence analysis (CCA) ordination plot shows the effect of physicochemical parameters of formation water on microbial community structures (genus level) in the CBM wells.

The relatedness among the microbial communities and the physicochemical parameters was done using CCA analysis. The CCA analysis at the genus level supports the microbial and physicochemical distinctiveness between the Producer and Marginal wells (Fig. 6). Axis 1 and Axis 2 account for 63.61% of the total variance. Physicochemical parameters like sodium, conductivity, TDS, fluoride, sulfate, and chloride were found to be related to *Fusibacter, Proteiniclasticum, Sulfospirillum, Youngiibacter, Coprothermobacter*, *Magnetospirillum*. Most of these bacteria are known for their hydrocarbon-degrading capabilities. Salts can modulate the hydrocarbon-degrading ability of microbes; recent findings stated higher expression of hydrocarbon-degradation-related genes with salt addition in slurry bioreactors^64^. A recent study has shown the relation between high bulk conductivities and TDS with enhanced mineral weathering, interlinked with the activities of hydrocarbon-degrading microbes in aquifers contaminated with hydrocarbons^65^. The strong relationship between chromium and bacteria suggests its importance in microbial metabolism in the ecosystem. Research has shown the role of trace metals, including chromium, in predicting methanogenic community structure and moderate concentrations of trace metals, which are essential for microbial functioning^66^. The CCA plot also reveals relatedness among members of *Methanothermobacter, Dictyoglomus, Syntrophothermus, Gelria, Rhizobium, Acetothermia, Azospira,* and *Desulfovibrio*. This indicates the presence of microbial syntrophy, which is essential for the process of methanogenesis^67^.

The CCA analysis shows an association of zinc, manganese, magnesium, copper, iron, and pH with microbial genera abundant in the Marginal wells. The analysis also revealed the relatedness among the genera *Methylobacterium, Rhodobacter, Reyranella, Methylococcus, Methylocystis, Hydrogenophaga,* and others. Copper and iron are widely studied to play a role in the methane oxidation mechanism of methanotrophic bacteria^68^. The analysis shows the crucial role of physicochemical parameters of the formation water in defining the microbial composition of the wells.

The findings of the study showed distinct microbial communities residing in the Producer and Marginal wells of the Bokaro region. Further, it emphasizes the role of physicochemical parameters of the formation water in framing distinct microbiomes. The results of the present study also reflect a higher diversity of degrading syntrophic, hydrogen-producing, and fermentative microbes in the Producer wells. Studies have supported the crucial role of these microbes in the process of methanogenesis^52,69^. Further, the results indicate a high abundance of various methylotrophs, which may be one of the significant parameters for decreased methane production in the wells. Previously, methylotrophs have been studied to significantly decrease methane production in different ecosystems^70,71^. Further exploration on methanotrophs is required to fully understand their role in the CBM production.

## Conclusion and future prospects

The enhancement of microbial-produced coal bed methane from marginal wells carries great environmental and economic benefits. Various factors like geography, coal rank, hydrology, and microbial composition influence the coal bed methane production. The present study revealed how microbial composition and environmental factors can significantly impact methane gas production in different CBM wells within a reservoir. The findings indicate a high abundance of methane-oxidizing bacteria in the Marginal wells, while Producer wells show a high abundance of hydrogen-producing and hydrocarbon-degrading microbes. A deeper knowledge of the microbial composition in CBM wells will help better strategize improvement in gas production in the marginal wells. The findings will help design optimized biostimulation and bioaugmentation jobs in the Marginal wells that can improve their CBM production efficiency. However, further investigation utilizing transcriptomics and proteomics analysis is required for a profound understanding of microbial metabolisms in CBM wells.

## Supplementary Information

Supplementary Information.

## Data Availability

All sequence data and metadata information are available through NCBI's Sequence Read Archive under Bioproject PRJNA945620. https://dataview.ncbi.nlm.nih.gov/object/PRJNA945620?reviewer=umren3f74e0m8kpim0tipck3vk.
