# Supplementary material for: Evaluation of microbial diversity in the formation water of the producer and marginal wells in bokaro coal field
Source: Sci Rep. 2024 Nov 28;14:29572. doi: 10.1038/s41598-024-61996-2 (PMC11605091; doi:10.1038/s41598-024-61996-2)
Supplement: Supplementary file 1 — Supplementary Information. [file 41598_2024_61996_MOESM1_ESM.pdf]

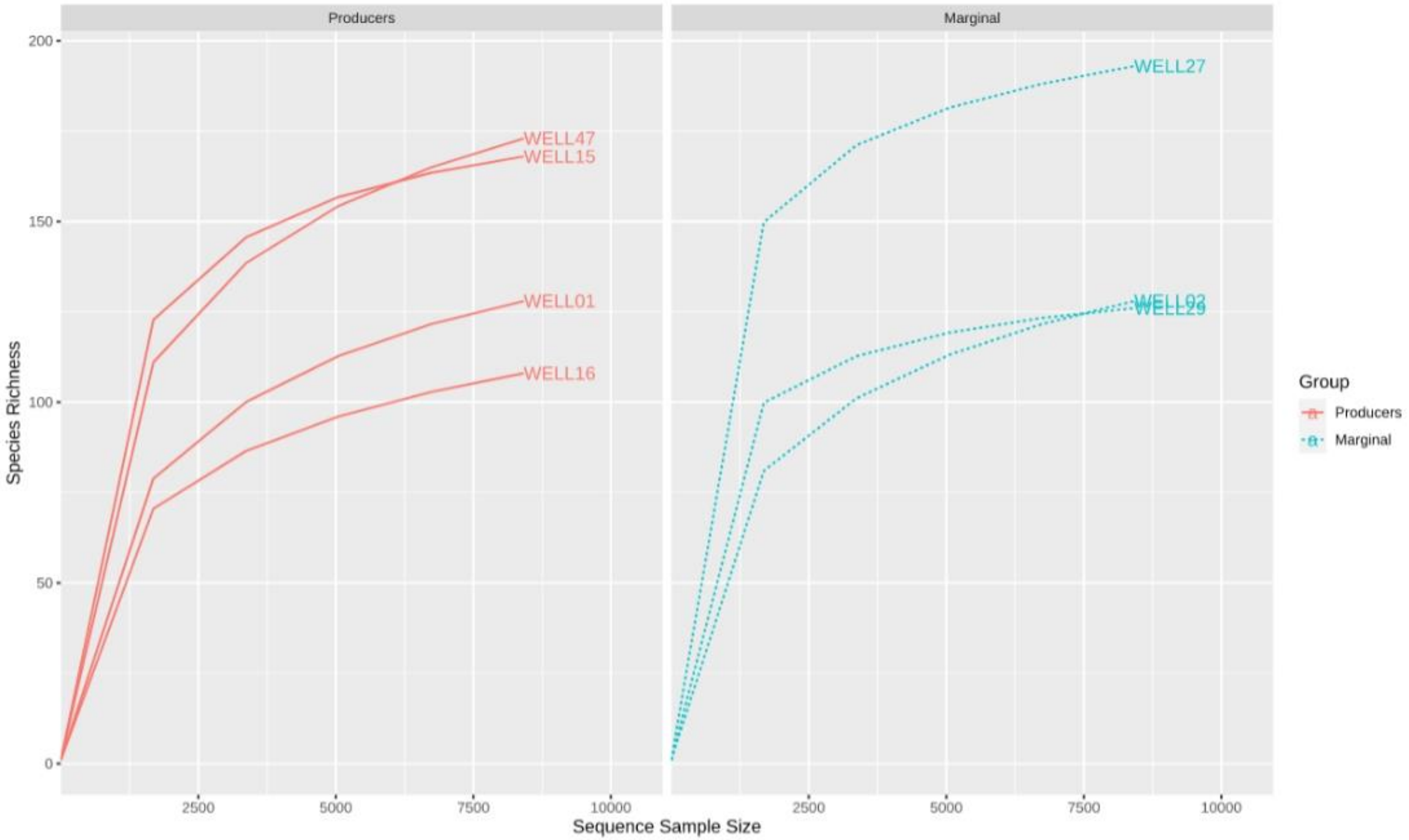

Figure S1 Rarefraction curve showing species richness in the samples

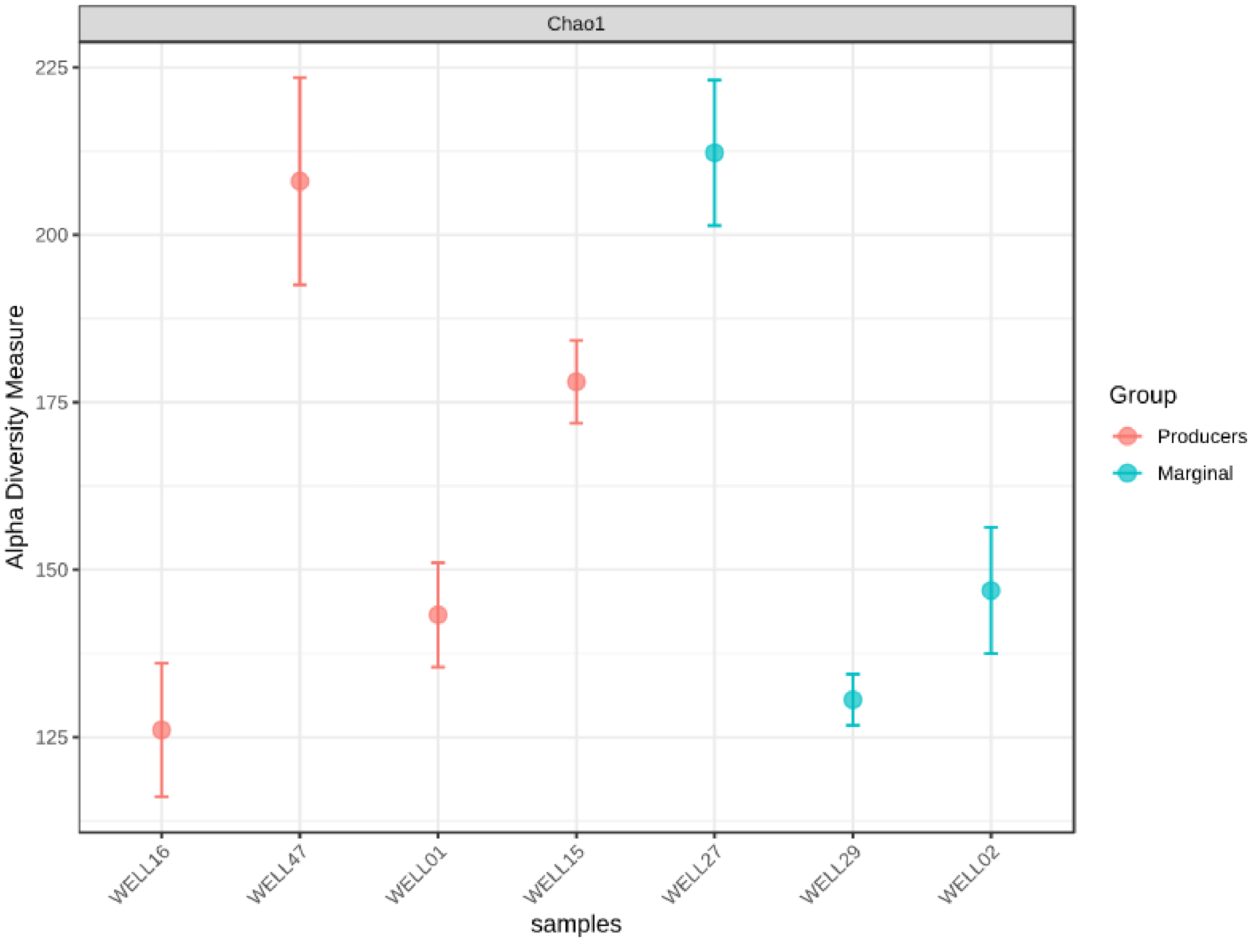

Figure S2 Alpha diversity analysis (Chao1).

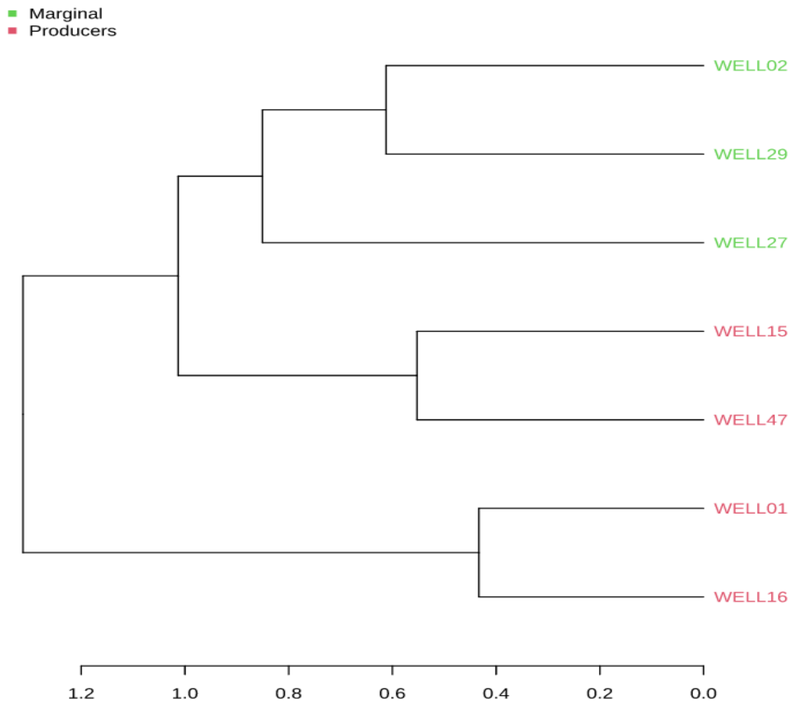

Figure S3 Dendrogram analysis at the genus level showing distinctness between the groups.
